# Identification of Control-Related Signal Path for Semi-Active Vehicle Suspension with Magnetorheological Dampers

**DOI:** 10.3390/s23125770

**Published:** 2023-06-20

**Authors:** Piotr Krauze

**Affiliations:** Department of Measurements and Control Systems, Silesian University of Technology, Akademicka 16, 44-100 Gliwice, Poland; piotr.krauze@polsl.pl

**Keywords:** all-terrain vehicle, magnetorheological damper, semi-active suspension system, vehicle dynamics model, Bouc–Wen model, identification of control-related signal path

## Abstract

This paper presents a method for the identification of control-related signal paths dedicated to a semi-active suspension with MR (magnetorheological) dampers, which are installed in place of standard shock absorbers. The main challenge comes from the fact that the semi-active suspension needs to be simultaneously subjected to road-induced excitation and electric currents supplied to the suspension MR dampers, while a response signal needs to be decomposed into road-related and control-related components. During experiments, the front wheels of an all-terrain vehicle were subjected to sinusoidal vibration excitation at a frequency equal to 12 Hz using a dedicated diagnostic station and specialised mechanical exciters. The harmonic type of road-related excitation allowed for its straightforward filtering from identification signals. Additionally, front suspension MR dampers were controlled using a wideband random signal with a 25 Hz bandwidth, different realisations, and several configurations, which differed in the average values and deviations of control currents. The simultaneous control of the right and left suspension MR dampers made it necessary to decompose the vehicle vibration response, i.e., the front vehicle body acceleration signal, into components related to the forces generated by different MR dampers. Measurement signals used for identification were taken from numerous sensors available in the vehicle, e.g., accelerometers, suspension force and deflection sensors, and sensors of electric currents, which control the instantaneous damping parameters of MR dampers. The final identification was carried out for control-related models evaluated in the frequency domain and revealed several resonances of the vehicle response and their dependence on the configurations of control currents. In addition, the parameters of the vehicle model with MR dampers and the diagnostic station were estimated based on the identification results. The analysis of the simulation results of the implemented vehicle model carried out in the frequency domain showed the influence of the vehicle load on the absolute values and phase shifts of control-related signal paths. The potential future application of the identified models lies in the synthesis and implementation of adaptive suspension control algorithms such as FxLMS (filtered-x least mean square). Adaptive vehicle suspensions are especially preferred for their ability to quickly adapt to varying road conditions and vehicle parameters.

## 1. Introduction

Controlled suspension systems of road vehicles that can adapt in real time to instantaneous road conditions are currently widely studied and developed. Here, semi-active and active suspension systems can be distinguished, where the former is favoured for its low energy consumption [1]. An MR (magnetorheological) damper is an example of a semi-active damper, in addition to electrorheological [2] and servo-valve dampers [3]. The MR damper exhibits a short response time, equal to 20 ms [4], and is therefore preferred for an instant reaction to road unevenness. However, the semi-active damper cannot add vibration energy to the mechanical system, but rather the dissipation of road-induced vibration energy is controlled. The force generated by MR dampers is strictly related to its piston motion, in contrast to the operation of an active force generator, which is much more independent of the controlled vehicle suspension. Thus, the operation of MR dampers included in a suspension system needs to be analysed in strict relation to the whole system and with the simultaneously generated road-induced excitation. This feature makes controlling MR dampers challenging and nontrivial.

Standard semi-active control schemes are generally divided into two parts. The lower control layer evaluates the appropriate control current based on the given desired force to make the MR damper generate a force as close to it as possible. The higher control layer corresponds to a vibration control mechanism and calculates the desired force that needs to be generated by the MR damper, as presented in [5]. The Skyhook algorithm introduced in [6] is known to be robust and widely used in vehicle suspension control. Its goal is to calculate the desired force that minimises the vibration of the vehicle body mass. Furthermore, different modifications of vibration control are proposed in the literature. The modified Skyhook algorithm [7] balances the Skyhook force and passive force, which maintains acceptable road holding during the Skyhook operation. The ADD algorithm (acceleration-driven damping) applied to a controlled vehicle suspension minimises the vertical acceleration of the vehicle body [8].

More complex control approaches have been further dedicated to the semi-active suspension. The Skyhook control can be combined with the fuzzy controller, as presented in [9]. Moreover, it was proposed in [10] that artificial neural networks can be used for the approximation of control parameters depending on different dominant excitation frequencies for a semi-active half-car suspension model. Iterative learning was proposed to suppress the vibrations of massive machinery components and was intended to be used with MR dampers [11]. The further integration of fuzzy controllers, neural networks, and optimisation algorithms was shown to be an effective option as a control method dedicated to a vehicle semi-active suspension system [12]. Here, an adaptive neuro-fuzzy inference system was applied for the modelling of MR damper behaviour, while the final controller was based on a fuzzy neural network accompanied by particle swarm optimisation and backpropagation as learning and training algorithms.

More efficient control algorithms take advantage of a model of the controlled process in addition to raw measurement data. Model predictive control is an example of such algorithms, and it can be applied to a semi-active suspension system with MR dampers, as was presented in [13]. Depending on the required complexity, which is mainly related to the number of considered degrees of freedom, different vehicle models are exploited in research studies. The classical quarter-car model [6], which is often used for theoretical analyses, describes the vertical dynamics of a quarter of the vehicle and consists of unsprung and sprung masses. Its extension, the half-car model [14], can additionally separately describe the front and rear wheels as well as the pitch motion of the vehicle body. Such a model is widely used in the literature: e.g., the analysis of different control strategies, including time delays and preview, was presented in [15]. A modification of the half-car model can be applied in order to describe a mountain bike rear suspension with a magnetorheological damper [16], where additional degrees of freedom map the longitudinal motion of the seat’s and the chain’s stay frame links. The full-car model, which exhibits seven degrees of freedom, allows one to map all major phenomena of vehicle vibration, including the heave, pitch, and roll motion of the vehicle body, as well as the vertical motion of each wheel. Such a model is widely used in the literature, e.g., for the validation of hybrid sliding mode control dedicated to the semi-active suspension system [17].

Models of more complex objects and processes, however, e.g., vehicles with MR dampers, are often difficult to fully identify, e.g., due to the requirement to generate the appropriate excitation or perform long experiments. In addition, such models need to be updated often, e.g., in the case of nonlinear systems and different working conditions. Thus, it is recommended in many cases to limit the identification of vehicle models to certain parts, frequency ranges, or applications, as in the case of the above-mentioned adaptive neuro-fuzzy inference system dedicated to an MR damper model [12]. Other researchers designed adaptive backstepping control dedicated to a simulated half-car model with MR dampers [18]. The resultant control system included two observers, which were used for the estimation of the dampers’ hysteresis internal states, and an algorithm for the adaptation of the parameters of the state-feedback control law. Another approach to the adaptive control of a semi-active suspension takes advantage of look-ahead road data, which influences control parameters during the vehicle ride. Such a solution was presented in [19] and was based on an LPV (linear-parameter-varying) algorithm integrated with a cruise controller, which was validated using the TruckSim simulation environment. The continuation of the study presented above was presented in [20], where a road adaptivity algorithm, which considers the vehicle velocity, road irregularities, and other look-ahead road information for the adaptation of LPV suspension controller parameters, is proposed. In order to improve the vehicle response to fishhook and double-lane-change manoeuvres, other authors proposed a model reference adaptive control, which is based on the AdaBoost algorithm, established using Lyapunov stability theory [21]. The proposed approach was shown to effectively predict rollover and improve the anti-rollover features of the vehicle.

The feed-forward FxLMS (filtered-x least mean square) represents a separate group of adaptive control algorithms, which tend to minimise the variance of a given error signal. Minimisation is achieved through the online tuning of an adaptive filter, which is simultaneously used to process a reference signal and generate a control signal for an actuator [22]. The FxLMS is also commonly computationally expensive, since it requires frequent operations of digital filtering. However, it can be implemented in different configurations: e.g., only a part of the algorithm is run during a selected period of time for the partial update mode [23]. The FxLMS and its modifications are widely used in different applications of vibration control. Methods of controlling the vibrations of the casing can be applied to mitigate the noise generated by devices, as reported in [24,25]. Another variant of the FxLMS, i.e., the narrowband FxLMS presented in [26], is especially desirable for the mitigation of harmonic disturbances. The algorithm can be dedicated to an inertial damper that acts as an actuator in the case of a reduction in vibrations that originate from an engine of a submarine vehicle and propagate to its hull through the engine mount [27]. Other authors proposed the FxLMS control of the active suspension of the vehicle to suppress the vertical force generated by an in-wheel switched reluctance motor of an electric vehicle [28], which, as a result, allowed a reduction in the acceleration of the sprung mass of the vehicle.

However, the application of the FxLMS for the control of semi-active dampers is more challenging because only the dissipation of energy is controlled and because the control signal (electric current or damping coefficient) is asymmetric and limited to positive values. A comparison of the FxLMS and the classical (non-filtered) LMS was designed for a seismically excited structure as a semi-active multi-variable adaptive controller [29] and validated on the basis of simulation results. Here, a simplified inverse model of the semi-active damper was applied for the conversion of the desired control force into the desired damping coefficient. Another solution based on a fast-convergence FxLMS algorithm was proposed in [30,31] for the control of a simulation model of a quarter-car hybrid suspension. The suspension included a low-frequency hydraulic actuator—SMA (spring mount adjustment)—and a high-frequency semi-active damper. The desired control force was generated by the FxLMS algorithm and further assigned, first to the SMA and second to the semi-active damper. Other examples of the FxLMS applied to the control of the classical suspension with MR dampers were presented in [32,33] and were dedicated to the half-car and full-car vehicle models, respectively. These solutions provided better vibration control than standard control algorithms, e.g., the Skyhook algorithm, and they potentially allow for rapid adaptation to varying parameters of vehicle suspension and to road conditions. The FxLMS applied to vehicles, in many cases, also takes advantage of a so-called preview signal, i.e., additional information available in advance about road unevenness or road obstacles located in front of the vehicle. Such information can be provided by different sensors, e.g., a single laser scanner or an algorithm of sensor fusion dedicated to mobile applications [34].

All applications of the FxLMS algorithm, including the above-mentioned studies, require the partial vehicle model, i.e., the dynamics of a control-related signal path, to be available. Such a model is critical in adaptive control algorithms (not limited to FxLMS), similar to using the knowledge about the gradient of the cost function in optimisation algorithms. It can improve the convergence of the algorithms or, in some cases, maintain convergence. In the case of active mechanical systems, the control-related signal path can be identified experimentally by generating wideband random excitation using the active actuator (the source of the disturbance is disabled) and by further analysing the system response. However, the discussed identification goal is not trivial in the case of a semi-active suspension, where vibration can be added to the mechanical system only by the disturbance, e.g., road-induced excitation, and is further dissipated in a controlled manner by the semi-active damper. Then, the response signal needs to be decomposed into the road-related component and a second group of components related to forces dependent on control currents and generated by different semi-active dampers. In such a case, the control-related signal path can be analytically or numerically derived based on the mathematical model of the controlled object, which is a common approach in the case of simulation-based studies. However, it is a troublesome method in the case of experimental studies and applications, because this again requires the identification of the entire model. Nevertheless, none of the above-mentioned studies related to semi-active systems addresses this problem of the identification of the control-related signal path, except for rare mentions and only general recommendations. Therefore, the method of identification applied in experiments and dedicated to a semi-active vehicle suspension is presented in the current study as the main contribution, and, in the author’s opinion, it responds to current needs related to adaptive semi-active control.

**Figure 1:**
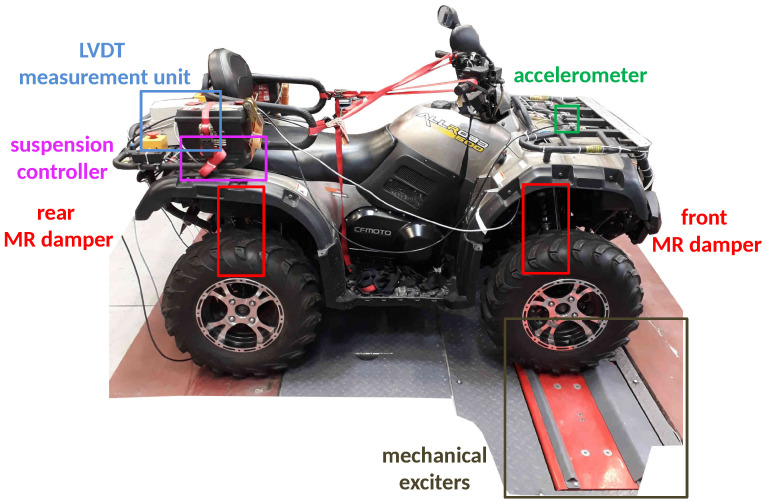
An experimental all-terrain vehicle with MR dampers subjected to harmonic excitation generated by mechanical exciters included in the vehicle diagnostic station.

The presented article is dedicated to an actual commercially available and real-size all-terrain vehicle (ATV) in which original shock absorbers were replaced with suspension MR dampers (see, e.g., [35]), presented in Figure 1. Numerous sensors available in the vehicle, e.g., an accelerometer, force sensors, suspension deflection sensors, and sensors of electric currents, which control the instantaneous damping parameters of MR dampers, allow for the comprehensive analysis of vibration propagation in the considered vehicle. Experiments were carried out using a modified diagnostic station that includes two mechanical exciters that generate the harmonic excitation of the selected frequency for the front wheels of the vehicle.

This article consists of five sections. In Section 2, dynamic models related to the experimental vehicle with MR dampers tested using the diagnostic station are defined, and the method of implementation of the vehicle vibration simulator is described. Section 3 describes the considered experimental conditions, including applied road-induced and control current excitations, as well as the preprocessing and preliminary analysis of measurement data obtained during experiments. In Section 4, the results of the identification of control-related models and the identified vehicle vibration model are presented and discussed. Furthermore, the influence of the vehicle load on control-related models is analysed in this section, while the main concluding remarks for the article are provided in Section 5.

## 2. Dynamic Vibration Model of Vehicle with MR Dampers and the Diagnostic Setup

**Figure 2:**
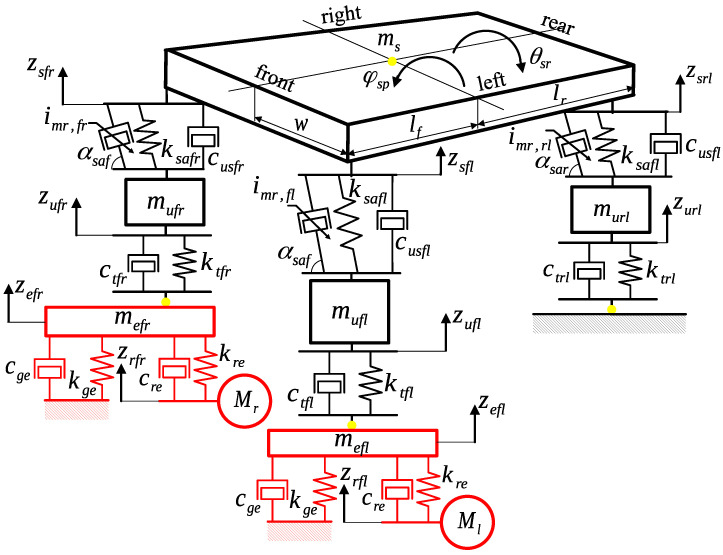
Mechanical representation of the vehicle vibration model (in black) including Bouc-Wen models of suspension MR dampers tested on a diagnostics station using two mechanical exciters (in red). Notations of vehicle directions: fr—front right, fl—front left, rr—rear right, rl—rear left.

The behaviour of the all-terrain vehicle with MR dampers during the discussed experiments is influenced by the complex structure of the vehicle design and the applied MR dampers. The presented experimental setup, which is described in detail within further parts of the manuscript, can be divided into three main elements, i.e., the diagnostic station with mechanical exciters, the all-terrain vehicle, and suspension MR dampers. To explain the dominant phenomena of vehicle behaviour, a mathematical description of the experimental setup was defined. It is visualised by a mechanical representation, presented in Figure 2, in the form of a vibrating mechanical system of lumped parameters, which consists of masses, springs, and dampers. The majority of the notations used within the presented article refer to the location of the considered component with the subscript ijb, where such phrases can take the following notation:

- *i*: *g*—Stationary ground of the diagnostic station; *r*—road-induced excitation signal or related to the diagnostic station; *e*—steel plate of the mechanical exciter; ge/re—interaction of the steel plate with the ground/exciter’s motor; *u*—vehicle wheel; *t*—vehicle tire; *s*—vehicle body; us—vertically oriented suspension; sa—tilted suspension shock absorber;
- *j*: *f*—Front; *r*—rear;
- *b*: *r*—Right; *l*—left.

Furthermore, for some cases presented in later parts of the manuscript, it is reasonable to use grouped notations that correspond to several physical quantities simultaneously. Thus, on the one hand, the notation $F_{safb}$ corresponds to the forces generated by the front shock absorbers and can be used in similar equations defined with respect to *b*. On the other hand, $k_{re}$ corresponds to the same stiffness parameter of the right and left mechanical exciters.

### 2.1. Road-Induced Excitation Generated by Mechanical Exciters

The front vehicle wheels are subjected to sinusoidal vibration excitation generated by the steel plates of two mechanical exciters, which are part of the classic vehicle suspension diagnostic station. Mechanical exciters are driven by three-phase electric motors through rotating shafts with an eccentric gear. This method of vibration excitation is an alternative to another type that is based on inertial exciters [36], where displacement is not the primary source of excitation. The control system of the diagnostic station was modified to control each of the mechanical exciters independently and simultaneously. Each of electric motors was controlled by a dedicated inverter, which allows the arbitrary selection of the excitation frequency in the range from 1.5 to 15 Hz. The operation of motor inverters was synchronised and supervised by an additional microprocessor controller. The description of the modified diagnostic station was previously presented in detail in [37].

The considered mathematical model of vibration exciters exhibits two degrees of freedom, which correspond to the vertical displacement $z_{efr}$ and $z_{efl}$ of the mass $m_{efr}$ and $m_{efl}$ of each exciter’s steel plate, respectively. For the purpose of modelling, it is assumed that each such plate is connected to the ground by a spring $k_{ge}$ and a viscous damper $c_{ge}$. Additionally, the steel plates are connected to driving eccentric gears by a spring $k_{re}$ and a viscous damper $c_{re}$. The synchronisation of both mechanical exciters is limited due to the limitations of motor power and the fact that the response of the vehicle to vibration excitation depends on its frequency. Thus, the road-induced excitation signals of the right and left vehicle wheels, denoted by $z_{rfr}$ and $z_{rfl}$, respectively, are defined independently with respect to time *t* as follows (b∈{r,l} as defined above):

(1) $$z_{rfb}=A_{rfb}\cos\left(2\pi f_{rfb}t+\delta_{rfb}\right),$$

where $A_{rfr}$, $f_{rfr}$, and $\delta_{rfr}$ and $A_{rfl}$, $f_{rfl}$, and $\delta_{rfl}$ denote the amplitude, frequency, and phase shift of the right and left vibration excitations, respectively. Vertical displacements of masses $m_{efr}$ and $m_{efl}$ are influenced from above by the vehicle’s front right and left wheels, described by tire forces $F_{tfr}$ and $F_{tfl}$, respectively (defined in Section 2.2). From below, the exciter plates are moved by forces $F_{efr}$ and $F_{efl}$, which depend on excitation signals $z_{rfr}$ and $z_{rfl}$, defined as follows:

(2) $$F_{efb}=-k_{re}\left(z_{efb}-z_{rfb}\right)-c_{re}\left({\dot{z}}_{efb}-{\dot{z}}_{rfb}\right)-k_{ge}z_{efb}-c_{ge}{\dot{z}}_{efb}.$$

### 2.2. Mathematical Description of the Vehicle Vibration Model

The vibration of the all-terrain vehicle is described in the presented manuscript by 7 degrees of freedom. Four degrees of freedom describe the vertical displacement of wheels, denoted by $z_{ufr}$, $z_{ufl}$, $z_{urr}$, and $z_{url}$, according to the notation defined above. Furthermore, three degrees of freedom are related to the pitch and roll angles of the vehicle body and the heave motion of its centre of gravity, denoted by $\varphi_{sp}$, $\theta_{sr}$, and $z_{s}$, respectively. They are related to the vehicle body mass $m_{s}$ as well as its pitch and roll moments of inertia, denoted by $I_{sp}$ and $I_{sr}$, respectively.

The considered mechanical representation of vehicle wheels consists of masses $m_{ufr}$, $m_{ufl}$, $m_{urr}$, and $m_{url}$; the corresponding tire stiffness parameters, denoted by $k_{tfr}$, $k_{tfl}$, $k_{trr}$, and $k_{trl}$; and tire viscous damping parameters, denoted by $c_{tfr}$, $c_{tfl}$, $c_{trr}$, and $c_{trl}$. As a result, the forces generated by consecutive tires influencing wheel masses are defined as follows:

(3) $$\begin{array}{cc} \; & F_{tfb}=-k_{tfb}\left(z_{ufb}-z_{efb}\right)-c_{tfb}\left({\dot{z}}_{ufb}-{\dot{z}}_{efb}\right),\\ \; & F_{trb}=-k_{trb}z_{urb}-c_{trb}{\dot{z}}_{urb}. \end{array}$$

The tire forces defined for the front wheels depend on vertical displacements of the mechanical exciters $z_{efb}$, while the rear wheels were stationary and located on the ground during laboratory experiments. Furthermore, the possibility of lifting the tires off the ground was taken into account by limiting forces $F_{tjb}$ to non-zero values only for negative values of tire deflection, defined as $\left(z_{ufb}-z_{efb}\right)$ and $z_{urb}$ in the case of front and rear wheels, respectively. Here, negative values of deflection are considered since the initial displacements of wheels are assumed to be zero, and they are further decreased by the gravitational field at the beginning of the simulation.

The suspension of the vehicle model is divided into four independent parts, where the shock absorbers of the front and rear suspensions are tilted at angles $\alpha_{saf}$ and $\alpha_{sar}$ relative to the ground, respectively. The vertical forces generated in these consecutive suspension parts, denoted by $F_{usfr}$, $F_{usfl}$, $F_{usrr}$, and $F_{usrl}$, are defined as follows:

(4) $$F_{usjb}=-c_{usjb}\left({\dot{z}}_{sjb}-{\dot{z}}_{ujb}\right)+g_{F_{saj}}\sin\left(\alpha_{saj}\right)\cdot F_{sajb},$$

where $c_{usfr}$, $c_{usfl}$, $c_{usrr}$, and $c_{usrl}$ denote the generalised viscous damping parameters of the selected suspension parts. The vertical displacements $z_{sjb}$ of different quarters of the vehicle body are evaluated based on $z_{s}$, $\varphi_{sp}$, and $\theta_{sr}$ as follows:

(5) $$\begin{array}{cc} \; & z_{sf(r/l)}=z_{s}-l_{f}\cdot \varphi_{sp}{\;}^{-}{/}_{+}\;w\cdot \theta_{sr},\\ \; & z_{sr(r/l)}=z_{s}+l_{r}\cdot \varphi_{sp}{\;}^{-}{/}_{+}\;w\cdot \theta_{sr}, \end{array}$$

where $l_{f}$ and $l_{r}$ define the longitudinal distance of the centre of gravity of the vehicle body to the front and rear points of the suspension attachment, respectively. The notation *w* corresponds to the lateral distance of the centre of gravity from the same attachment points.

The forces generated by consecutive shock absorbers, denoted by $F_{safr}$, $F_{safl}$, $F_{sarr}$, and $F_{sarl}$, are dependent on their relative deflections $z_{sajb}$:

(6) $$\begin{array}{cc} \; & F_{sajb}=-k_{sajb}\cdot z_{sajb}+F_{\mathrm{mr},jb}\left(v_{sajb}\right),\\ \; & z_{sajb}=g_{z_{saj}}\cdot \left(z_{sjb}-z_{ujb}\right). \end{array}$$

**Figure 3:**
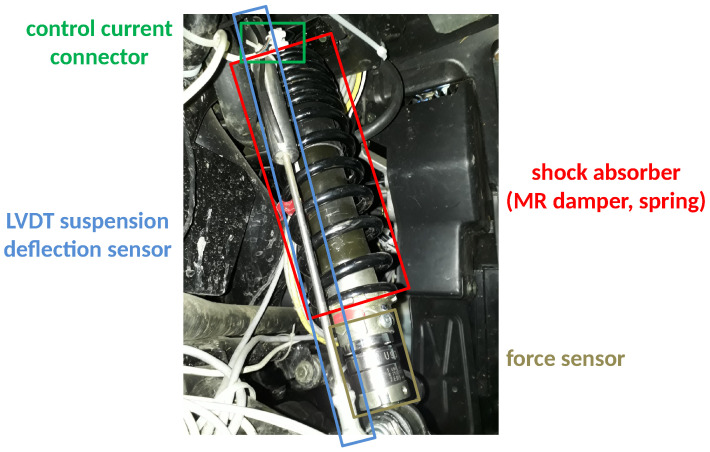
Elements of the measurement and control system installed in the experimental vehicle: shock absorber, consisting of an MR damper supplied with control current and steel coil spring, and suspension force and LVDT deflection sensors.

The notation $v_{sajb}={\dot{z}}_{sajb}$ corresponds to the velocity related to a selected displacement quantity. These additional notations, in addition to acceleration $a=\ddot{z}$, are defined in order to clarify the description of the Bouc–Wen model or certain parts of the further analysis related to experimental results and measurement signals. The stiffnesses of the springs built into the consecutive shock absorbers are denoted by $k_{safr}$, $k_{safl}$, $k_{sarr}$, and $k_{sarl}$, while the forces generated by consecutive suspension MR dampers are denoted by $F_{\mathrm{mr},fr}$, $F_{\mathrm{mr},fl}$, $F_{\mathrm{mr},rr}$, and $F_{\mathrm{mr},rl}$. The conversions of the relative displacements of the suspension and the forces, denoted by $g_{z_{saj}}$ and $g_{F_{saj}}$, respectively, result from the mechanical advantage of the lever of the double wishbone suspension design, as presented in Figure 3. As can be seen, the MR damper manufactured by Lord Corporation and connected to the controller is built into the suspension spring within the shock absorber. Additionally, the LVDT suspension sensor and the force sensor that were installed using dedicated mechanical adapters can be observed.

### 2.3. Bouc–Wen Model of MR Dampers

The MR damper can be compared to a standard shock absorber, with the difference being that it is filled with MR fluid, which consists of magnetisable particles suspended in a carrier fluid, often mineral oil [1,38]. The MR fluid flowing through gaps located in the damper’s piston is subjected to a magnetic field induced by electric coils. Then, these particles reorganise into chain-like structures along the lines of the magnetic field, and they locally impede the flow of the MR fluid. On the macroscopic scale, it results in an increase in the damping coefficient of the MR damper. The operation of the MR damper and its nonlinear behaviour are commonly analysed using characteristics showing the force generated by the MR damper with respect to its relative piston velocity. Force saturation phenomena can mainly be observed for higher piston velocities, while hysteresis loops are commonly dominant for lower velocities.

The model of the MR damper is a MISO system (multiple-input single-output) with two inputs: the control current and piston velocity; the damper force is the single output. Numerous models of MR dampers have been presented in the literature that try to explain the dominant phenomena of the behaviour of MR dampers. The saturation of the generated force is generally described in the literature using a Bingham model [39] that includes the Coulomb friction force as the key component, accompanied by viscous damping. Further models extend it with the additional analysis of a gas accumulator located at the end of the damper cylinder, such as Gamota–Filisko [40] or other such models that take into account the noticeable stiffness in the MR damper response. The mechanical representation of the Gamota–Filisko model includes a series connection of the Bingham model, Kelvin–Voight body, and Hooke body models. The heuristic Tanh model is a composition of phenomenological parts, mainly related to viscous damping and, optionally, stiffness components, and is a black-box model. The Tanh model is based on the hyperbolic tangent function, which is similar to the shape of force–velocity characteristics obtained during experiments. Other approaches represented by the Tanh model have been discussed in numerous studies; e.g., identification results for the Tanh model were presented in [1,41]. Furthermore, the Tanh model was implemented and applied in simulations and experiments in the inverse form dedicated to control applications, as was presented in [32].

An extended analysis presented in the literature is dedicated to the hysteresis loop observed in the damper’s force–velocity characteristics. The Bouc–Wen model and its extension—the Spencer model [40]—take advantage of the Bouc–Wen component, which accurately maps the nonlinear shape of force–velocity characteristics. The Bouc–Wen model is favoured for its good match to the operation of the MR damper, which is maintained in a wide range of vibration frequencies. It is especially important in the considered process, where the frequency of the mechanical exciter is increased from 0 up to 15 Hz and back to the idle state in a relatively short time. Here, the Bouc–Wen model is mathematically described for each part of the considered vehicle suspension by the following set of equations with respect to the relative velocity of the shock absorber $v_{sajb}$:

(7) $$\begin{array}{cc} \; & {\dot{p}}_{sajb}=-\alpha_{bw,jb}\cdot |v_{sajb}|\cdot p_{sajb}\cdot |p_{sajb}{|}^{n_{bw}-1}-\beta_{bw,jb}\cdot v_{sajb}\cdot {\left|p_{sajb}\right|}^{n_{bw}}+A_{bw,jb}\cdot v_{sajb},\\ \; & F_{\mathrm{mr},jb}=-\gamma_{bw,jb}p_{sajb}-c_{bw,jb}v_{sajb}, \end{array}$$

where the former is a nonlinear first-order differential equation defining the dynamics of the hysteresis displacement $p_{sajb}$, and the latter is a formula for the output MR damper force. Most of the model parameters are related to the selected part of the vehicle suspension due to the fact that they directly or indirectly depend on the corresponding control current $i_{\mathrm{mr},jb}$.

The parameters of the Bouc–Wen model applied in the current study were estimated based on experimental data discussed as part of another publication that is related to a trajectory control algorithm proposed for a vibrating screen with a semi-active suspension [42]. The same type of MR damper as that installed in the experimental vehicle was separately examined during identification experiments using a material test system [32]. A set of experiments were carried out for different combinations of sinusoidal excitation subjected to a damper piston of selected amplitudes and frequencies (1.5 Hz, 15 mm; 6 Hz, 5 mm; 20 Hz, 2 mm), as well as for several values of constant control current (0A, 0.37A, 0.5A, 0.63A, 0.8A, 1.15A, 1.4A).

The model parameters were initially estimated simultaneously for three excitation frequencies separately for each current value by minimising the mean-squared error calculated between the measured and simulated forces. It was concluded based on preliminary results that for the automotive application considered, the number of above-mentioned parameters, defined as $\alpha_{bw}$, $\beta_{bw}$, $A_{bw}$, $n_{bw}$, $\gamma_{bw}$, and $c_{bw}$, is redundant and can be limited by assuming the following dependencies:

(8) $$\begin{array}{cc} \; & \alpha_{bw}=\lambda_{bw}\cdot \epsilon_{bw},\\ \; & \beta_{bw}=\lambda_{bw}\cdot \left(1-\epsilon_{bw}\right),\\ \; & A_{bw}=\lambda_{bw}. \end{array}$$

Further, $\gamma_{bw}$, $\lambda_{bw}$, and $c_{bw}$ were made dependent on the control current $i_{\mathrm{mr}}$ through third-degree polynomials, as follows:

(9) $$\gamma_{bw}=\sum_{l=0}^{3}\gamma_{bw,l}\cdot i_{\mathrm{mr}}^{l}\;,\;\;\;\lambda_{bw}=\sum_{l=0}^{3}\lambda_{bw,l}\cdot i_{\mathrm{mr}}^{l}\;,\;\;\;c_{bw}=\sum_{l=0}^{3}c_{bw,l}\cdot i_{\mathrm{mr}}^{l}\;.$$

The values of all parameters of the Bouc–Wen model applied in the following part of the manuscript are listed in Table 1.

**Figure 4:**
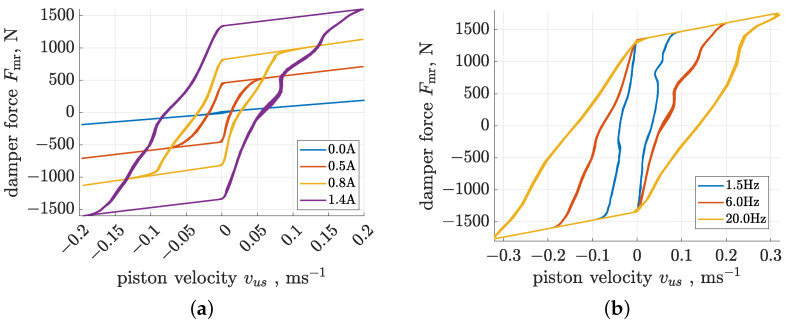
Force–velocity characteristics of the implemented Bouc–Wen damper model demonstrated for selected configurations of sinusoidal piston excitation and control current ${\hat{i}}_{\mathrm{mr}}$: (**a**) model responses obtained for sinusoidal excitation at 6 Hz, 5 mm, and several control currents; (**b**) model responses obtained for control current equal to 1.4 A and different configurations of sinusoidal excitation.

Exemplary responses of the implemented Bouc–Wen model determined for the selected configurations are compared in Figure 4. The first set of characteristics were evaluated for selected control currents and piston displacement excitation with a frequency of 6 Hz and an amplitude of 5 mm. The presented model describes three dominant features of MR damper behaviour, i.e., the force saturation phenomenon as well as the size of hysteresis loops and force levels dependent on the control current. Small undulations of the characteristics result from actual measurements of piston excitation signals. The second set of characteristics generated for a control current equal to 1.4 amperes represent the behaviour of the Bouc–Wen model for different frequencies of piston excitation. Here, the model reflects the actual damper behaviour, where the size of hysteresis loops is directly proportional to the excitation frequency.

### 2.4. Implementation of the Vehicle Vibration Simulator

The resultant 9 ordinary differential equations (grouped according to previously mentioned notations), constituted based on the formulas presented above, describe the dynamic vehicle vibration model and the diagnostic station:

(10) $$\begin{array}{cc} \; & m_{efb}{\ddot{z}}_{efb}=F_{efb}-F_{tfb},\;\;\;/\;\times 2\;\mathrm{with}\;\mathrm{respect}\;\mathrm{to}\;b\\ \; & m_{ujb}{\ddot{z}}_{ujb}=F_{tjb}-F_{usjb},\;\;\;/\;\times 4\;\mathrm{with}\;\mathrm{respect}\;\mathrm{to}\;jb\\ \; & m_{s}{\ddot{z}}_{s}=F_{usfr}+F_{usfl}+F_{usrr}+F_{usrl},\\ \; & I_{sp}{\ddot{\varphi }}_{sp}=-l_{f}\cdot \left(F_{usfr}+F_{usfl}\right)+l_{r}\cdot \left(F_{usrr}+F_{usrl}\right),\\ \; & I_{sr}{\ddot{\theta }}_{sr}=-w\cdot \left(F_{usfr}+F_{usrr}\right)+w\cdot \left(F_{usfl}+F_{usrl}\right), \end{array}$$

The complete vehicle model system is based on 24 state variables, where the first 18 variables are generalised coordinates and their derivatives, defined based on Equation (10). Further, 4 variables describe hysteresis displacements $p_{sajb}$ of each Bouc–Wen model included in the vehicle model. The additional 2 state variables denoted by $z_{\mathrm{res}}$ and ${\dot{z}}_{\mathrm{res}}$ are related to the implementation of a harmonic oscillator, which is further defined by Equation (16) in Section 4.3. As a result, the complete vector *X* of state variables used for the vehicle simulator is defined as follows:

(11) $$\begin{array}{cc} X=[ & z_{efr},z_{efl},z_{ufr},z_{ufl},z_{urr},z_{url},z_{s},\varphi_{sp},\theta_{sr},\\ \; & {\dot{z}}_{efr},{\dot{z}}_{efl},{\dot{z}}_{ufr},{\dot{z}}_{ufl},{\dot{z}}_{urr},{\dot{z}}_{url},{\dot{z}}_{s},{\dot{\varphi }}_{sp},{\dot{\theta }}_{sr},\\ \; & p_{safr},p_{safl},p_{sarr},p_{sarl},z_{\mathrm{res}},{\dot{z}}_{\mathrm{res}}{]}^{T}. \end{array}$$

The vector *U* of the input variables, which is defined below, consists of 8 elements. It includes four currents controlling the corresponding suspension MR dampers $i_{\mathrm{mr},jb}$, as well as road-induced excitations $z_{rfb}$ and their derivatives ${\dot{z}}_{rfb}$ generated by mechanical exciters:

(12) $$U=[i_{\mathrm{mr},fr},i_{\mathrm{mr},fl},i_{\mathrm{mr},rr},i_{\mathrm{mr},rl},z_{rfr},z_{rfl},{\dot{z}}_{rfr},{\dot{z}}_{rfl}{]}^{T}.$$

The presented differential equations of all modelled components were reformulated in the following generalised nonlinear state-space form to implement the vehicle simulator in the C programming language:

(13) $$\dot{X}=F_{X}\left(X,U\right).$$

The main nonlinearity, which is described within the vector of functions $F_{X}$ included in Equation (13), is introduced into the vehicle simulator by MR damper models since they are included in the feedback loops by the damper piston velocities $v_{sajb}$. However, it can be concluded that another significant nonlinearity is due to the possibility of the detachment of vehicle wheels, taken into account in the case of tire forces $F_{tfb}$, defined in Equation (3). The above-presented state-space equation is solved numerically using the fourth-order Runge–Kutta–Fehlberg method, including an adaptive stepsize approach, implemented as a part of the GNU scientific library.

## 3. Preprocessing and Analysis of Experimental Data

**Figure 5:**
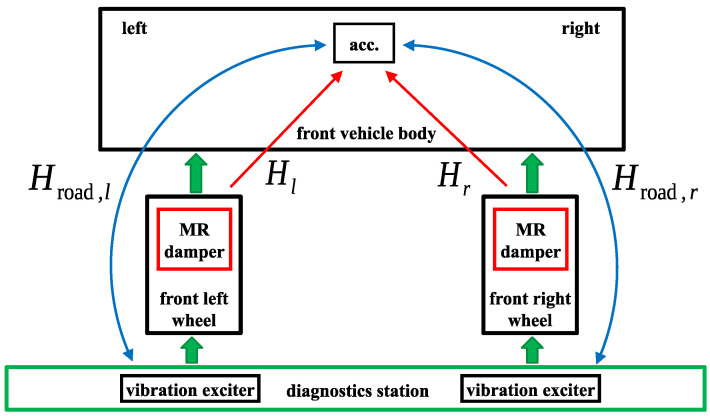
General vehicle model including different disturbance- and control-related signal paths.

The control-related signal path is defined from a selected actuator to a so-called error signal. The error measurement signal is taken for a part of the object whose vibration is to be mitigated by the FxLMS or another type of adaptive algorithm. For the purpose of future studies, the vertical acceleration of the middle part of the front vehicle body, denoted by $a_{sf}$, was selected as the error signal, which is assumed to be potentially mitigated. As presented in the block diagram in Figure 5, it can be stated that four excitation signals (with respect to the identification perspective, not the implementation referred to in Section 2.4) mainly contribute to the error signal $a_{sf}$. The two independent road-induced sinusoidal excitations are generated by mechanical exciters and propagate through the structure of the vehicle, including the constant damping of the MR dampers of the rear suspension, as described by the signal paths $H_{\mathrm{road},r}$ and $H_{\mathrm{road},l}$. The forces generated by the front MR dampers $F_{\mathrm{mr},fb}$, which are controlled by the corresponding electric currents $i_{\mathrm{mr},fb}$, are assumed to be two additional excitations. In summary, the control-related signal paths defined from forces $F_{\mathrm{mr},fr}$ or $F_{\mathrm{mr},fl}$ to the error signal $a_{sf}$, further denoted by $H_{r}$ or $H_{l}$, respectively, are to be identified.

The results and descriptions of experiments presented in further parts of the manuscript are compiled and intertwined with the descriptions of simulations. Thus, in order not to confuse the reader and clearly indicate the origin of certain results, the following notation is introduced:

- *x*—General usage of quantity *x*, valid for both simulation and measurement results;
- $\hat{x}$—Quantity *x* evaluated based on simulation results;
- $\overset{˘}{x}$—Quantity *x* evaluated on the basis of the measurement results.

### 3.1. Identification Procedure for Control-Related Signal Paths

The task of $H_{r}$ and $H_{l}$ identification is not trivial, despite the fact that both input and output measurement signals are available. First, the outputs of both signal paths simultaneously contribute to the resultant signal $a_{sf}$, which is additionally influenced by the remaining dominant components related to the road-induced harmonic excitation. Secondly, force input signals of $H_{r}$ and $H_{l}$ are strongly correlated with the road-induced excitation, specifically its harmonics, and consequently are mutually correlated, leading to an ill-conditioned identification algorithm. Thus, the proposed identification procedure consists of the following phases related to the preparation, experiments, and data post-processing, which are described in detail in further parts of the article:

- Confirmation that the sinusoidal road-related excitation generated using the diagnostic station is appropriate for the considered identification, the selection of its frequency equal to 12 Hz, and the selection of a wideband low-frequency control current signal with a bandwidth equal to 25 Hz;
- The implementation of experiments for different amplitudes and average values of MR damper control currents;
- The filtering of harmonic components of the road-induced excitation from the analysed force and acceleration measurement signals and the compensation of the influence of suspension springs;
- The simultaneous identification of $H_{r}$ and $H_{l}$ in the frequency domain based on previously preprocessed signals.

The identified transfer functions, which are determined in the frequency domain and not in the form of parametric models, provide complete information about the discussed control-related signal paths, and such knowledge can be used in vibration control algorithms. The identification of control-related models in the form of digital filters and their integration into adaptive control algorithms will be the direct consequence and the subject of further research. The approximation of control-related models using a limited structure of digital filters commonly deteriorates the accuracy of models. Here, several identification methods can be proposed, e.g., the instrumental variable (IV) method, as presented in [43], which is recommended for autoregressive-moving-average models if the disturbance cannot be described as white noise that is filtered with the same autoregressive model.

### 3.2. Measurement and Control System of the Experimental Vehicle

**Figure 6:**
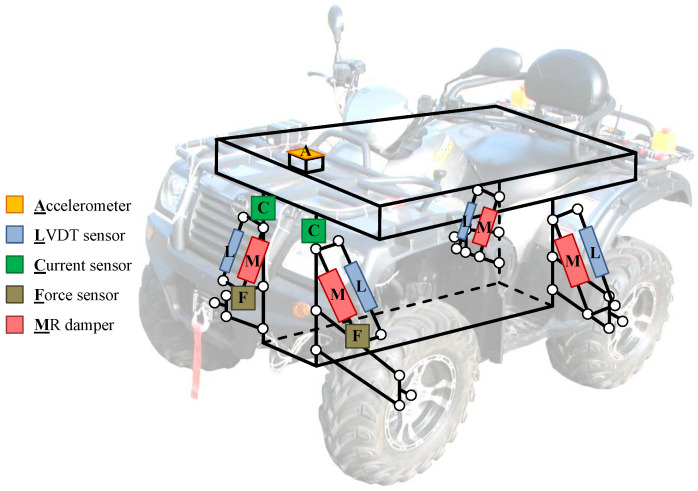
Sensors and actuators of the measurement and control system available in the experimental vehicle and used during presented experiments.

The real-sized all-terrain experimental vehicle with MR dampers (presented in Figure 1) is equipped with a measurement and control system that includes several sensors used for vehicle vibration and motion measurements dedicated to the identification procedure, as presented in Figure 6. In addition to the sensors considered in the presented study, additional sensors are also available in the vehicle, as presented in the author’s previous publications [35,37]: multiple accelerometers measuring the vibration of the vehicle body and wheels, an IMU module (inertial measurement unit), and wheel rotational speed sensors.

For all experimental results presented in the past and currently analysed, the control current is generated for each suspension MR damper by an independent peripheral measurement and control unit supervised by a main suspension controller. The main controller communicates with all peripheral units through a CAN bus. Here, the operation of the peripheral units were slightly modified in order to generate predefined random low-frequency control current signals, which is discussed in more detail in Section 3.3. The experiments presented were carried out for four variants of random currents, generated in a bandwidth of 25 Hz and with a distribution close to Gaussian. As a result, control currents generated for consecutive experimental cases take the following average values: 0.12, 0.36, 0.59, and 0.78 amperes; the corresponding standard deviations of the random components are 0.08, 0.15, 0.24, and 0.32 amperes.

The measurement signals taken from the sensors used in the presented study were acquired by an additional measurement controller with a sampling frequency equal to 10 kHz. Further, these signals were calibrated, filtered using a digital 8th-order Butterworth low-pass filter with a cut-off frequency equal to 100 Hz, decimated to a sampling frequency equal to 1 kHz, and prepared for the identification procedure. For the purpose of the discussed identification procedures, the following sensors were mainly used:

- Current transducers (LTS 6-NP) configured for a measuring range equal to ±2 A, manufactured by LEM, separately measuring electric currents controlling the front suspension MR dampers (MR dampers located in the rear parts of the vehicle suspension were unpowered during experiments—passive mode of operation); these measurements were used for the identification of the vehicle model, not the control-related signal paths;
- Force transducers (U93) with a measuring range equal to ±5 kN, manufactured by HBM, separately measuring forces generated by front shock absorbers;
- LVDT (linear variable differential transformer) sensors with a measuring range equal to 60 mm, manufactured by Peltron, separately measuring suspension deflections of front shock absorbers;
- Three-axis MEMS (microelectromechanical system) low-noise accelerometer (ADXL356) configured for a measuring range equal to ±10 g (gravitational acceleration), manufactured by Analog Devices, attached to and measuring the acceleration of the middle front part of the vehicle body.

**Figure 7:**
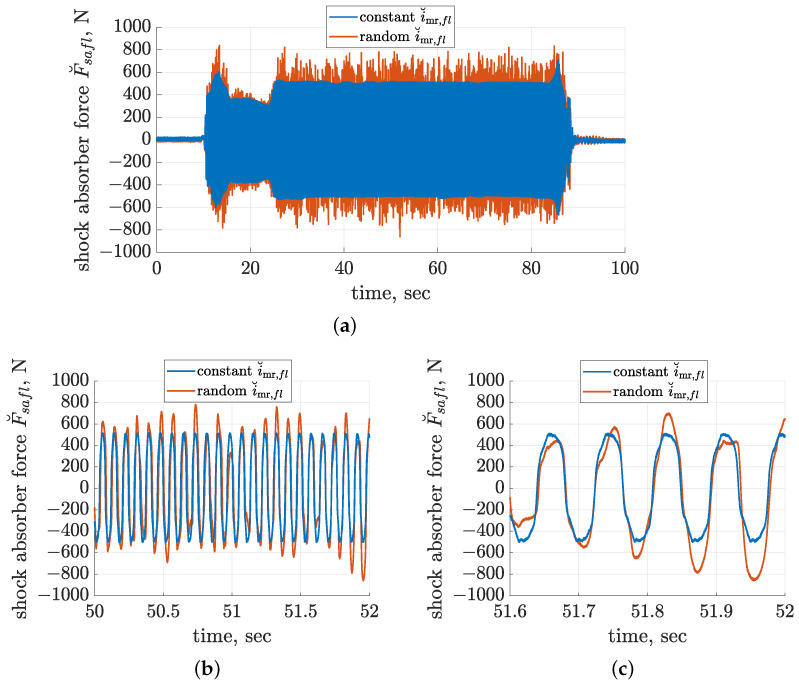
Comparison of time diagrams of force generated by the front left shock absorber ${\overset{˘}{F}}_{safl}$ related to constant and random control currents ${\overset{˘}{i}}_{\mathrm{mr},fl}$ evaluated based on experimental results and related to the second configuration with an average control current equal to 0.36 A and a standard deviation equal to 0.15 A: (**a**) the whole experiment, (**b**) time range of 50–52 s, and (**c**) time range of 51.6–52 s.

Each experiment carried out using the dedicated diagnostic station in the given configuration consisted of the following phases: starting the electric motors that drive the mechanical exciters up to frequency equal to 15 Hz, stabilising the operation frequency for 15 s, decreasing the frequency down to the target 12 Hz, stabilising the frequency and continuously synchronising both mechanical exciters for 60 s, stopping the electric motors, and entering idle time in order to avoid their overheating. The consecutive phases of operation are clearly visible in Figure 7a, where the comparison of the forces ${\overset{˘}{F}}_{safl}$ measured for the left front shock absorber is presented (the force offset was neglected in the presented case). The compared time diagrams were obtained for two experimental cases related to a constant electric current equal to 0.36 A controlling the MR dampers or its extension, including an additional random component with a standard deviation equal to 0.15 A. For the random control current, the force response of the MR damper is also random and clearly not smooth, and it propagates to the vehicle body accelerometer and is further exploited within the identification.

During start-up, the resonance of the vehicle suspension occurs, which results in a maximum amplitude of force equal to 600 or 800 N for a constant or random control current, respectively. Further, the force amplitude is stabilised for the target 12 Hz vibration frequency, and in the case of the constant control current, it is approximately equal to 500 N. This is clearly shown in the zoomed-in time diagrams in Figure 7b,c, where, in the case of the random control current, the measured force varies within a range of approximately ±800 N. Finally, mechanical exciters stop, while suspension resonance occurs again when the force amplitude increases to approximately 700 or 800 N for the constant or random case, respectively.

The controllers of mechanical exciters and motor inverters take advantage of their own independent set of sensors measuring the rotational speed and, indirectly, the frequency of each exciter, and these sensors also measure the synchronisation of both exciters in the form of the time gap occurring between their rotations. These measurements are commonly used for the validation of the operation of mechanical exciters. Additionally, measurements of the exciters’ frequencies were used to identify the vehicle model.

### 3.3. Modulation Effect of Road-Related and Control-Related Excitation Signals

**Figure 8:**
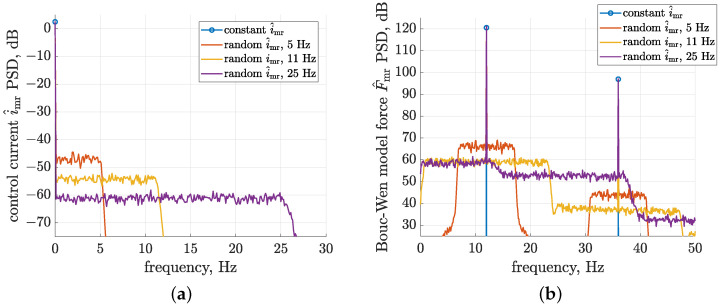
Simulated effect of Bouc–Wen MR damper model subjected to piston displacement road-related excitation ${\hat{z}}_{sa}$ with 1 mm amplitude and 12 Hz frequency modulated by a constant or a wide-band low-pass random control current ${\hat{i}}_{\mathrm{mr}}$ with bandwidth of 5, 11, or 25 Hz: (**a**) PSD frequency characteristics of different cases of control current ${\hat{i}}_{\mathrm{mr}}$; (**b**) PSD frequency characteristics of corresponding force responses of Bouc–Wen model ${\hat{F}}_{\mathrm{mr}}$.

The reliable identification of control-related signal paths requires their inputs, i.e., the MR damper forces $F_{\mathrm{mr},fb}$, to be sufficiently excited within the selected frequency, which is defined in the presented study up to 25 Hz. As a consequence, a control scheme was implemented for the MR dampers, which comes down to the generation of a harmonic component of the force response modulated by a random electric current supplied to the damper. Such behaviour demonstrated in Figure 8 based on simulation results recalls features of the classical modulation effect, where the carrier frequency is accompanied by two sidebands that come from a single baseband signal. From the Bouc–Wen model perspective, it can be explained that the modulation mechanism is valid mainly due to the fact that the model parameters, which are dependent on the control current $i_{\mathrm{mr}}$, are multiplied by the hysteresis displacement $p_{sajb}$, which is dependent on the relative velocity of the piston.

The following results were obtained for the Bouc–Wen model subjected to a sinusoidal piston displacement excitation ${\hat{z}}_{sa}$ with a 1 mm amplitude and a frequency equal to 12 Hz, which are close to the experimental conditions. The force response was modulated by a wide-band low-frequency random control current ${\hat{i}}_{\mathrm{mr}}$ with different bandwidths, i.e., 5, 11, or 25 Hz, and with a Gaussian distribution. The average value and standard deviation of these simulated control currents refer to one of the previously mentioned experimental cases and are equal to 0.36 A and 0.15 A, respectively.

The first set of presented PSD (power spectral density) characteristics were evaluated in the frequency range from 0 to 30 Hz using the *pwelch* function available in the Matlab environment implementing Welch’s averaged periodogram method [44]. The characteristics were calculated for a constant current or a set of the above-defined random control currents, i.e., baseband signals according to the modulation mechanism. Three cases of random control currents defined for bandwidths up to 5, 11, or 25 Hz were compared. The PSDs of consecutive cases decrease due to Parseval’s identity since, regardless of the different bandwidths, the same signal variance was maintained. The resultant force responses of the Bouc–Wen model presented as PSD frequency characteristics from 0 to 50 Hz indicate that the corresponding control currents were transferred to the vicinity of the carrier frequency, equal to 12 Hz, and to the further odd harmonics of the fundamental frequency, equal to 36 and 60 Hz. The dominant contribution of only odd harmonics is due to the fact that damper piston excitation is processed by the odd force–velocity characteristics exhibited by the MR damper, as was discussed in Section 2.3.

The random control current with a frequency bandwidth up to 5 Hz is consequently transferred due to modulation to the frequency range from 7 to 17 Hz, while the second current signal is transferred from a bandwidth equal to 11 Hz to the frequency range from 1 to 23 Hz. Baseband signals transferred to the vicinity of the third 36 Hz harmonics exhibit a lower PSD since the power of these harmonics is lower. In the case of baseband signals with a bandwidth greater than the carrier frequency, e.g., the third case corresponding to a bandwidth equal to 25 Hz, the damper force response for all frequencies results from the overlapping of mainly two different and neighbouring sidebands.

For the frequency range from 0 to 12 Hz, the resultant PSD of the third case is comparable to the case of a baseband signal with a bandwidth equal to 11 Hz. However, for higher frequencies, a decrease in PSD can be noticed, as can be expected based on the current control PSD characteristics since the higher-frequency neighbouring sideband of the lower PSD does not fully compensate for the loss of PSD caused by the greater bandwidth of the baseband signal. Thus, it can be suggested that the best combination for identification carried out in frequency bands from 0 to 25 Hz could be achieved for the road-related excitation of a frequency equal to 12.5 Hz and a control current with the same bandwidth. However, it was validated that the final configuration with a harmonic frequency equal to 12 Hz and a current signal bandwidth equal to 25 Hz was sufficient for the selected identification task, as discussed in Section 4. The need for the configuration of peripheral measurement and control units for a wider range of experiments and the time consumption of such a reconfiguration, as well as the previous validation of mechanical exciters for a frequency equal to 12 Hz, were also the main reasons for this selection.

### 3.4. Filtering of Road-Induced Excitation from Measurement Data

The applied sensors measure the force generated by shock absorbers, including suspension MR dampers and springs. However, the identification of control-related signal paths is limited to suspension MR dampers only. Thus, the compensation of the spring forces is treated as the first phase of the presented filtering procedure. The compensation is carried out on the basis of the measured force, as defined by the inverse of Equation (6). As a consequence, the spring force that is added for each suspension part is estimated using a previously identified and known stiffness parameter and the shock-absorber deflection measured by the corresponding LVDT sensor.

The second step of filtering is to exclude the road-induced harmonic component from the considered force excitation signals and the acceleration response signal. As a result of filtering, a random wide-band component is left in these signals, whose generation was discussed in Section 3.3. The front suspension MR dampers were controlled by independently generated random electric currents. Thus, the random components included in the force signals were highly uncorrelated, in contrast to the filtered harmonic component, which is common for all mentioned signals. The low correlation between input signals is further favourable for the identification of multiple-input models, and it allows one to avoid an ill-conditioned identification procedure.

The cross-correlation of two selected signals is generally assessed by using the correlation coefficient, which, in extreme cases, is equal to 1 for the same signals and equal to 0 for two independent random signals. The filtering of harmonic components applied to force measurement signals allowed us to decrease the cross-correlation coefficients for the consecutive experimental cases previously discussed from values of 0.37, 0.42, 0.28, and 0.19 to the following corresponding values: 0.06, 0.06, 0.14, and 0.14. For some identification cases, it is justified to additionally analyse the maximum values of the cross-correlation function evaluated over different lags, which, in this case, decreases the corresponding values of 0.71, 0.73, 0.56, and 0.75 to the following values: 0.07, 0.08, 0.14, and 0.14. The initial cross-correlation coefficient of the force signals is lower for higher control currents, which could be explained by greater overloads and, consequently, the nonlinearities of the vehicle suspension revealed in these experimental cases. Thus, decreasing the cross-correlation by filtering the harmonic components is also more efficient for lower control currents.

Generally, two approaches to the analysis and processing of measurement signals can be distinguished in the literature. Processing performed in the time domain is carried out in cases where the signal is processed online or computing resources are insufficient to carry out complex processing in the frequency domain. Here, a least Lp-norm filter that processes a one-dimensional signal within a time window could be an interesting example of a solution that allows us to attenuate impulses included in measurement data [45]. Other solutions dedicated to the compensation of harmonic disturbance can utilise a bank of notch filters, e.g., as presented in [46]. The second approach related to the frequency domain is based on FFT (fast Fourier transform) and is widely used to determine different frequency components in vibration measurements [47], commonly in cases where all measurement data are available offline. Otherwise, time–frequency analysis is recommended when the change in frequency characteristics needs to be analysed in time for subsequent fragments of the signal, e.g., in the case of diagnostics of vibrating systems [48].

**Figure 9:**
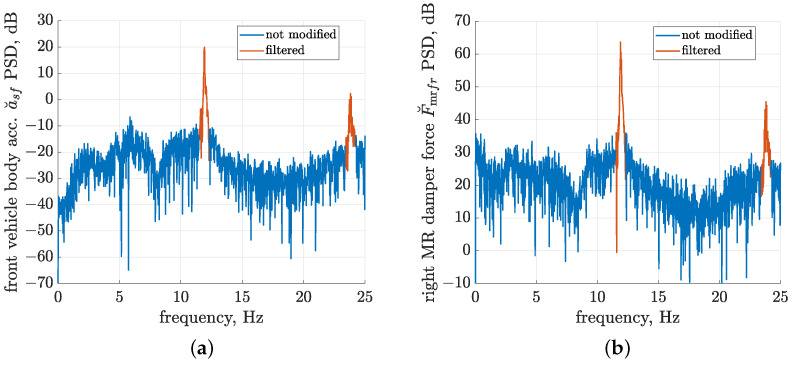
Frequency characteristics of PSDs (calculated based on FFTs, which were used for filtering of road-induced harmonic components) evaluated for experimental results of the second configuration of average control current equal to 0.36 A and standard deviation equal to 0.15 A: (**a**) PSD of front vehicle body acceleration ${\overset{˘}{a}}_{sf}$; (**b**) PSD of right MR damper force ${\overset{˘}{F}}_{\mathrm{mr}fr}$. Frequency ranges of filtering are marked in red.

The presented study takes advantage of the fact that all measurement data were available in advance. As a consequence, offline filtering was carried out in the frequency domain, and this allowed the precise removal of harmonics by calculating the fast Fourier transform (the *fft* function implemented in the Matlab environment) and by zeroing the selected frequency ranges marked in Figure 9. It presents PSDs evaluated based on the corresponding FFTs, which were calculated for acceleration ${\overset{˘}{a}}_{sf}$ and force ${\overset{˘}{F}}_{\mathrm{mr}fr}$ measurement signals in the frequency range from 0 to 25 Hz. Consequently, inverse FFTs were calculated, and the resultant filtered signals were obtained in the time domain for further processing. The preliminary analysis of measurement data allowed us to optimise the filtering frequency ranges, which were defined as the ±0.4 Hz neighbourhood of the consecutive harmonics of a frequency equal to 11.9 Hz. The slight decrease in the actual average frequency of road-induced harmonics generated by mechanical exciters, compared to the nominal 12 Hz, is largely due to the algorithm for the synchronisation of vibration exciters. In the selected periods of time, one of the mechanical exciters was temporarily slightly slowed down by decreasing its frequency in order to maintain sufficient synchronisation between exciters during each experiment.

An additional question could arise as to why the identification of signal paths was not applied to each MR damper separately by modulating the force of only one selected MR damper and simultaneously supplying the second damper with a corresponding mean value of the constant control current. In the author’s opinion, the simultaneous control of both MR dampers is closer to the target application, where all suspension MR dampers are used at the same time for vibration control. Furthermore, different realisations of random control currents allow the maintenance of low cross-correlations between wide-band components of forces generated by both MR dampers. As a consequence, the influence of both MR dampers on vehicle body acceleration can be efficiently decoupled. Finally, the similar control of both parts of the vehicle suspension could allow one to maintain comparable loads on both vibration exciters and, consequently, ensure their more stable operation. However, the influence of the selected experimental cases on the power consumption and efficiency of the vibration exciters was not analysed in the presented study.

## 4. Identification of Control-Related Signal Paths and Vehicle Vibration Model

The following part of the manuscript begins with a presentation of the results and analysis dedicated to control-related models identified in the frequency domain for different configurations of control currents. It is supported by an introductory analysis of linear dependencies revealed between the inputs and output of the analysed system carried out based on coherence frequency characteristics. The method and results of identification show, among others, the influence of increasing the mean values of control currents on the identified models. Next, the parameters of the vehicle vibration model discussed in Section 2 are tuned in order to fit the control-related models obtained in the simulations to the experimental results. Finally, the influence of vehicle load variation on control-related models, which are crucial for adaptive control algorithms, is analysed based on the vehicle model implemented.

### 4.1. Coherence of Force Excitation and Response Signals

The coherence estimate indicates how well the inputs $F_{\mathrm{mr},fr}$ and $F_{\mathrm{mr},fl}$ are linearly related to the output $a_{sf}$ for consecutive frequencies and can be modelled as a linear system, where a value of 0 indicates no linear relation and 1 confirms a fully linear relation. The analysis of coherence was performed using the *mscohere* function implemented in the Matlab environment, which estimates the magnitude-squared coherence function according to the method presented in [49]. Here, a variant of coherence dedicated to multiple-input systems needs to be applied according to the following definition:

(14) $$\begin{array}{cc} \; & C_{Fa}\left(f\right)=\\ \; & {\left[\begin{array}{c} P_{F_{\mathrm{mr},fr}a_{sf}}^{*}\left(f\right)\\ P_{F_{\mathrm{mr},fl}a_{sf}}^{*}\left(f\right) \end{array}\right]}^{T}{\left[\begin{array}{cc} P_{F_{\mathrm{mr},fr}F_{\mathrm{mr},fr}}\left(f\right) & P_{F_{\mathrm{mr},fr}F_{\mathrm{mr},fl}}\left(f\right)\\ P_{F_{\mathrm{mr},fl}F_{\mathrm{mr},fr}}\left(f\right) & P_{F_{\mathrm{mr},fl}F_{\mathrm{mr},fl}}\left(f\right) \end{array}\right]}^{-1}\left[\begin{array}{c} P_{F_{\mathrm{mr},fr}a_{sf}}\left(f\right)\\ P_{F_{\mathrm{mr},fl}a_{sf}}\left(f\right) \end{array}\right]\frac{1}{P_{a_{sf}a_{sf}}\left(f\right)} \end{array}$$

where $P_{xx}$ denotes the power spectral density of signal *x*, and $P_{xy}$ denotes the cross-power spectral density of signals *x* and *y* estimated based on Welch’s averaged periodogram method [44]. The symbol $P_{xx}^{*}$ denotes the complex conjugate of $P_{xx}$. Coherence is calculated element-wise according to Equation (14) with respect to consecutive frequencies.

**Figure 10:**
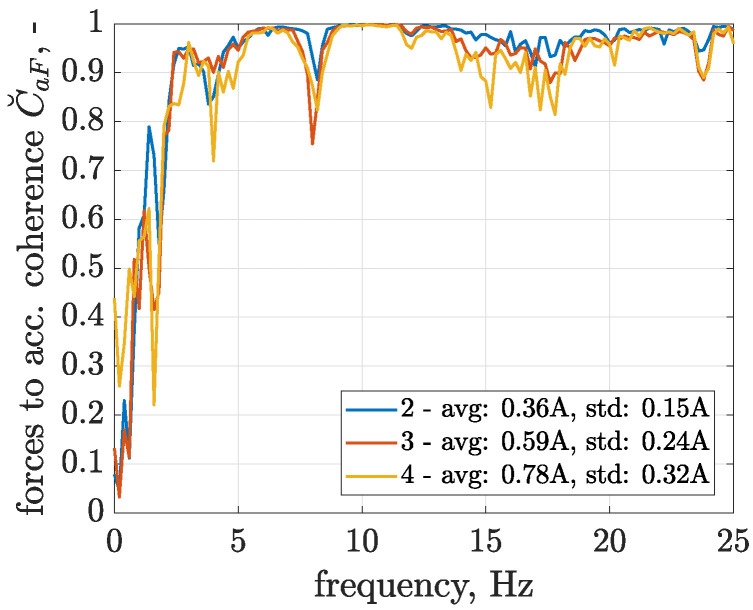
Comparison of frequency characteristics of coherence defined from the inputs ${\overset{˘}{F}}_{\mathrm{mr},fr}$ and ${\overset{˘}{F}}_{\mathrm{mr},fl}$ to the output ${\overset{˘}{a}}_{sf}$, evaluated for three configurations of average values and standard deviations of control current ${\overset{˘}{i}}_{\mathrm{mr}}$.

The coherence frequency characteristics calculated for the selected configurations of control currents are presented in Figure 10 and indicate that coherence is close to 1 for most of the analysed frequencies starting from 2.0 up to 25 Hz, while for the second configuration, the trusted frequency range could even be extended to as low as 1.4 Hz. Such an observation also confirms results of identification reported later, where significant resonance peaks are revealed for frequencies starting from 2 Hz. It can be stated that coherence is slightly decreased with increasing average values of control currents, and consequently, it can be concluded that such control configurations increase the overall nonlinearity of control-related signal paths. The decrease in coherence is especially visible for lower frequencies starting from 1.2 to 1.9 Hz and for selected frequencies in a higher range from 13.5 to 19 Hz.

### 4.2. Experimental Results of Identification of Control-Related Signal Paths

Data sets of processed force and acceleration measurement signals with a length equal to 48 s corresponding to the most stable operation of mechanical exciters were selected for each configuration of control currents. The estimation of the transfer function evaluated in the frequency domain for both signal paths, denoted by $H_{r}$ and $H_{l}$, was carried out using the *tfestimate* function implemented in the Matlab environment according to the algorithm presented in [50]. Here, its variant dedicated to multiple-input systems was applied according to the following formula:

(15) $$\begin{array}{cc} \; & H_{f}\left(f\right)=\frac{P_{a_{sf}F_{\mathrm{mr},fr}}\left(f\right)\cdot P_{F_{\mathrm{mr},fl}F_{\mathrm{mr},fl}}\left(f\right)-P_{a_{sf}F_{\mathrm{mr},fl}}\left(f\right)\cdot P_{F_{\mathrm{mr},fl}F_{\mathrm{mr},fr}}\left(f\right)}{P_{F_{\mathrm{mr},fr}F_{\mathrm{mr},fr}}\left(f\right)\cdot P_{F_{\mathrm{mr},fl}F_{\mathrm{mr},fl}}\left(f\right)-P_{F_{\mathrm{mr},fr}F_{\mathrm{mr},fl}}\left(f\right)\cdot P_{F_{\mathrm{mr},fl}F_{\mathrm{mr},fr}}\left(f\right)},\\ \; & H_{l}\left(f\right)=\frac{P_{a_{sf}F_{\mathrm{mr},fl}}\left(f\right)\cdot P_{F_{\mathrm{mr},fr}F_{\mathrm{mr},fr}}\left(f\right)-P_{a_{sf}F_{\mathrm{mr},fr}}\left(f\right)\cdot P_{F_{\mathrm{mr},fr}F_{\mathrm{mr},fl}}\left(f\right)}{P_{F_{\mathrm{mr},fr}F_{\mathrm{mr},fr}}\left(f\right)\cdot P_{F_{\mathrm{mr},fl}F_{\mathrm{mr},fl}}\left(f\right)-P_{F_{\mathrm{mr},fr}F_{\mathrm{mr},fl}}\left(f\right)\cdot P_{F_{\mathrm{mr},fl}F_{\mathrm{mr},fr}}\left(f\right)}, \end{array}$$

where the conjugate of the cross-power spectral density $P_{yx}^{*}=P_{xy}$. Transfer functions are calculated element-wise according to Equation (15), similarly to the previously defined coherence function, with respect to consecutive frequencies.

**Figure 11:**
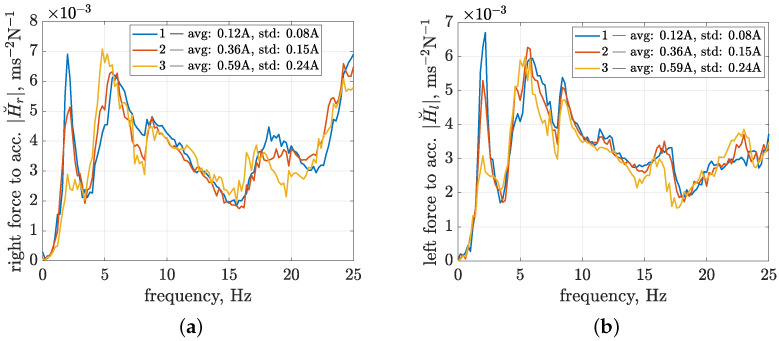
Comparison of frequency characteristics of absolute transfer functions of control-related signal paths evaluated based on experimental results ($|{\overset{˘}{H}}_{r}|$,$|{\overset{˘}{H}}_{l}|$) and related to three configurations of average values and standard deviations of control current ${\overset{˘}{i}}_{\mathrm{mr}}$, related to (**a**) the right suspension part; (**b**) the left suspension part.

The identification results are presented for the first three configurations of control currents in Figure 11 in the form of absolute frequency characteristics of the estimated transfer functions. The frequency characteristics of the last configuration, which correspond to an average control current equal to 0.78 A and a standard deviation equal to 0.32 A, are comparable to the third configuration. Thus, they are omitted to improve the clarity of the presentation. The analysis is focused on a frequency range up to 25 Hz since it is commonly exploited in the literature when discussing the influence of vibration on vehicles and passengers and dealing with corresponding methods of suspension control.

The frequency characteristics reach values of absolute transfer functions of up to 7 × 10${}^{-3}$ ms${}^{-2}$N${}^{-1}$. Several frequency ranges can be distinguished for the presented frequency characteristics, which are related to certain resonance peaks. Varying control currents obtained for different experimental configurations have the most decisive influence on the first resonance peak, located close to 2 Hz. Thus, it is suspected to be related to the damping of the front suspension, where a greater value of the average control current and, consequently, suspension damping causes the first resonance peak to be suppressed. The second resonance peak, located from approximately 4 to 7.5 Hz, appears to be a combination of two components, where the first one, located in the frequency range from 4 to 5.5 Hz, is clearly dependent on the control configuration, as it increases with greater values of average control currents and their corresponding standard deviations.

The remaining frequency ranges of the absolute transfer functions appear to be less dependent on the control configuration, except for minor changes visible for higher frequencies. For the transfer function of the right suspension, an inversely proportional influence of the control current, as previously revealed for the first resonance peak, can be noticed within the frequency range from 17.5 to 21.5 Hz. In the case of the left part of the vehicle suspension, additional stiffening caused by the increase in the control current can be noticed for frequencies from 21 to 23.5 Hz. The clearest differences, which are visible when comparing models obtained for the front and left suspension parts and are not discussed above, can be observed with the amplification of characteristics located in the frequency range from 21.5 Hz for the right MR damper and the frequency range defined from 14.5 to 18 Hz for the left MR damper.

Furthermore, by comparing the absolute transfer functions obtained for the experimental and simulation results, further discussed in Section 4.3, it was concluded that the minima visible for higher frequencies in the characteristics of both the right and left suspension parts at 15.5 Hz and 17.5 Hz, respectively, are related to the resonance frequencies of the corresponding vehicle wheels, mainly their masses and stiffness parameters.

### 4.3. Identification of Vehicle Vibration Model

The vehicle vibration model was obtained as part of the presented research in order to explain selected phenomena revealed by the identified models of control-related signal paths and to discuss additional scenarios related to vehicle exploitation that are difficult to validate experimentally at the current stage. The vehicle model is still under development, and it will be extended in future research studies dedicated, among others, to suspension control algorithms. However, the current stage of model development was considered satisfactory to be applied in the presented study.

The below-presented results of vehicle model identification are dedicated to the second configuration of suspension control, corresponding to an average electric current equal to 0.36 A and a standard deviation equal to 0.15 A. First, simulated road-induced excitation signals were generated according to Equation (1). These excitations were obtained on the basis of the measured frequencies of the mechanical exciters and measurements of the phase shift between them to perform simulations closely corresponding to the selected experimental case. Second, the resulting road-induced excitations were synchronised with measurements of electric currents supplied to the MR dampers. Finally, the four road-induced and control-induced signals mentioned above were fed into the vehicle model. As a result, simulated signals of forces generated by the MR damper models and the acceleration of the middle vehicle body were processed according to the same procedure as the experimental results to obtain control-related models.

The potential future development of the vehicle model that is coupled with the diagnostic station and mechanical exciters can be carried out from several aspects. For instance, the following identification of the vehicle model currently does not takes into account resonance peaks visible within the frequency range from 21.5 Hz for the transfer function dedicated to the right MR damper and within the frequency range from 14.5 to 18 Hz in the case of the left MR damper. However, a major exception is that the control-related signal paths of the implemented model do not include the second resonance peak visible within the frequency range from 4 to 7.5 Hz, especially the part that is independent of the control current.

A preliminary analysis of the simulation results obtained for different control configurations suggests that this resonance peak can be caused by the vibration of the rear wheels, which are coupled with the rear vehicle body by a stiffened rear suspension. Although rear MR dampers were not supplied during experiments, such an instantaneous effect of a suspension lock could occur if the deflection of the rear suspension were forced in a direction that is not fully in line with the axis of the shock absorbers. The simulation of the vehicle model conducted for the rear MR dampers supplied with a high control current, which practically led to a suspension lock, induced the discussed resonance peak but with an insufficient amplitude. Therefore, it was decided that this aspect requires future studies and the installation of additional sensors in the rear part of the vehicle, while, for the purpose of the presented study, the resonance peak discussed was emulated by an additional harmonic oscillator described by the following differential equation:

(16) $${\ddot{z}}_{\mathrm{res}}+2\zeta_{\mathrm{res}}\omega_{\mathrm{res}}{\dot{z}}_{\mathrm{res}}+\omega_{\mathrm{res}}^{2}z_{\mathrm{res}}=g_{\mathrm{res}}\cdot g_{F_{saf}}\sin\left(\alpha_{saf}\right)\cdot (F_{safr}+F_{safl}),$$

where the equivalence $a_{\mathrm{res}}={\ddot{z}}_{\mathrm{res}}$ is further applied in the article and denotes the acceleration of the harmonic oscillator response. The harmonic oscillator is excited by vertical components of the forces generated by the MR damper models. The symbols $\omega_{\mathrm{res}}$ and $\zeta_{\mathrm{res}}$ denote the undamped angular frequency of the oscillator and its damping ratio, respectively.

In conclusion, the goal of vehicle model identification was to obtain the best fit of the model response to the experimental results with respect to the absolute values of the transfer functions identified for the right and left control-related signal paths within the frequency range from 1 to 25 Hz. This statement can be described by the following expression:

(17) $$\begin{array}{c} \mathrm{identification}\;\mathrm{goal}:\\ \;\;\;|{\overset{˘}{H}}_{r}\left(f\right)|,|{\overset{˘}{H}}_{l}\left(f\right)|<\mathrm{curve}\;\mathrm{fitting}>|{\hat{H}}_{r}\left(f\right)|,|{\hat{H}}_{l}\left(f\right)|\;,\;\mathrm{for}\;f\in \langle 1\;\mathrm{Hz},25\;\mathrm{Hz}\rangle , \end{array}$$

taking into account the above-mentioned exceptions related to the selected resonance peaks and frequency ranges. The absolute transfer functions $|{\hat{H}}_{r}|$ and $|{\hat{H}}_{l}|$ are evaluated based on ${\hat{a}}_{sf}=a_{sf}+a_{\mathrm{res}}$, which includes the response of the vehicle model defined in Section 2 and the response of the additional harmonic oscillator defined in Equation (16), respectively. The vehicle model parameters were tuned in several stages, while the final values are listed in Table 2.

The first stage consisted of the preliminary estimation of parameters based on previous knowledge and known geometrical dependencies. Starting from mechanical exciters, each of the steel plates is supported by a steel coil spring $k_{ge}$, which is further supported on the ground, and by a flexible steel plate with equivalent stiffness $k_{re}$ connected to an eccentric gear driven by the rotating shaft. Thus, the resultant stiffness $k_{ge}+k_{re}=240,000\;{\mathrm{Nm}}^{-1}$ was estimated in the idle state of the exciter by loading the steel plate with a known weight and measuring its deflection. The resultant stiffness of the bending steel plate $k_{re}$ was estimated based on the results of simulated strength tests carried out in the FreeCAD design environment after the prior preparation of the simulation model of this plate. The amplitude of the vertical displacement of the eccentric gear was measured during its manual rotation. Further conclusions drawn from measurements indicated that the resonance frequency of mechanical exciters should be close to 8.5 Hz, while additional assumptions about a low damping ratio allowed the estimation of the resultant vibrating masses $m_{efr}$ and $m_{efl}$ of exciters.

The second set of parameters are related to the vehicle wheels, whose masses $m_{ujb}$ were weighed, and preliminary values of the corresponding tire stiffness parameters $k_{tjb}$ were estimated by loading them with a known weight and measuring the resultant tire deflection. The third set of parameters are related to the vehicle body, where initial values of the sprung mass $m_{s}$ and moments of inertia $I_{sp}$ and $I_{sr}$ were estimated based vehicle approval documents dedicated to the experimental off-road vehicle and based on the vehicle’s physical dimensions. The distances that describe the locations to mount the shock absorbers on the vehicle body, determined by $l_{f}+l_{r}$ and 2w, were estimated based on geometric parameters obtained from the approval documents. Certain values of $l_{f}$ and $l_{r}$ describing the centre of gravity of the vehicle body part were determined by measuring the load of each wheel on the ground.

The next set of model parameters considered are dedicated to the shock absorbers and their mounting in the vehicle. The stiffness parameters of steel coil springs built into the front and rear shock absorbers, denoted by $k_{safb}$ and $k_{sarb}$, respectively, were previously measured using the MTS (material testing system) after shock absorber disassembly. Additional coefficients—such as $g_{z_{saj}}$ for converting relative suspension displacements, $g_{F_{saj}}$ for converting forces generated by front and rear shock absorbers, and angles $\alpha_{saj}$ for describing the nominal inclination of shock absorbers in the suspension—derive from the double wishbone suspension design, as discussed in Section 2. The values of these parameters were calculated for a model of the double wishbone suspension implemented based on information available in the approval documents. Finally, the parameters $\omega_{\mathrm{res}}=2\pi f_{\mathrm{res}}$, $\zeta_{\mathrm{res}}$, and $g_{\mathrm{res}}$ of the harmonic oscillator were adjusted to the second resonance peak revealed in Figure 11, assuming a resonance frequency $f_{\mathrm{res}}=5.5\;\mathrm{Hz}$.

**Figure 12:**
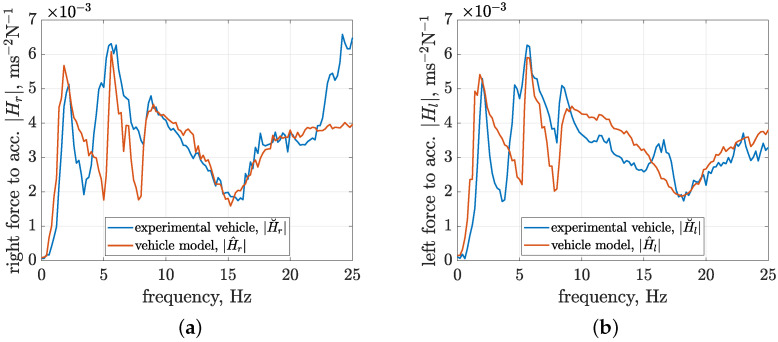
Frequency characteristics of absolute transfer functions of control-related signal paths evaluated based on experimental results ($|{\overset{˘}{H}}_{r}|$,$|{\overset{˘}{H}}_{l}|$) and related to the second configuration with an average control current equal to 0.36 A and a standard deviation equal to 0.15 A, compared to transfer functions evaluated for the simulated responses of the identified vehicle model including mechanical exciters ($|{\hat{H}}_{r}|$,$|{\hat{H}}_{l}|$) related to (**a**) the right suspension part; (**b**) the left suspension part.

The second stage of identification was dedicated to the final tuning of the model parameters, which was carried out manually and through an automated approach, resulting in model responses fitted to the experimental absolute transfer functions, as presented in Figure 12. The main goal of this stage was the adjustment of parameters dedicated to viscous damping related to the mechanical exciters $c_{ge}$ and $c_{re}$, vehicle tires $c_{tjb}$, and suspension $c_{usjb}$. Additionally, the tire stiffness parameters of the front wheels were adjusted based on the conclusions made in Section 4.2 that the minima visible in the experimental characteristics of front and rear absolute transfer functions at frequencies of 15.5 and 17.5 Hz, respectively, are related to the wheels of the vehicle. In conclusion, the presented results of vehicle model identification were considered satisfactory for the purpose of the presented study.

### 4.4. Influence of Vehicle Load on Control-Related Signal Paths

**Figure 13:**
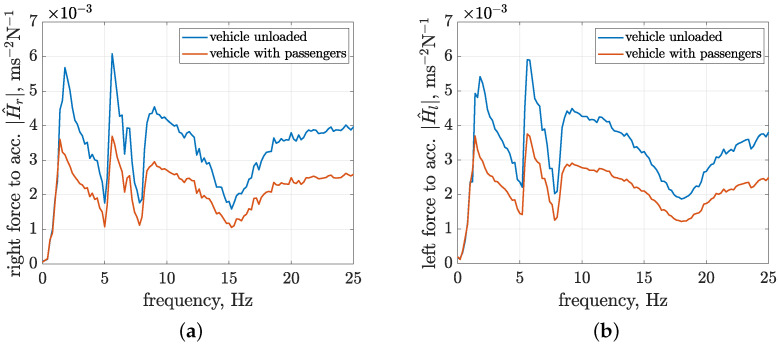
Comparison of frequency characteristics of absolute transfer functions of control-related signal paths $|{\hat{H}}_{r}|$ and $|{\hat{H}}_{l}|$, evaluated for the simulated responses of the identified vehicle model unloaded or with passengers, related to (**a**) the right suspension part; (**b**) the left suspension part.

**Figure 14:**
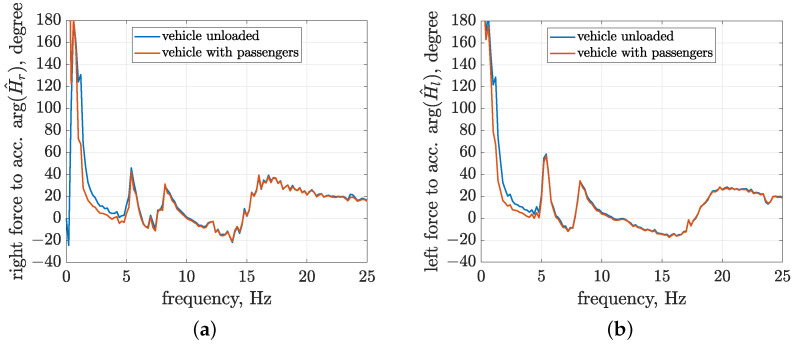
Comparison of frequency characteristics obtained for phase shift of transfer functions dedicated to control-related signal paths $\arg({\hat{H}}_{r})$ and $\arg({\hat{H}}_{l})$ and evaluated for the simulated responses of the identified vehicle model unloaded or with passengers, related to (**a**) the right suspension part; (**b**) the left suspension part.

The most frequently changed vehicle parameter is probably its weight. It is especially noticeable in the case of smaller off-road vehicles, such as the experimental vehicle under consideration, which is designed to carry up to two persons. For the estimated mass of the vehicle body, equal to 300 kg, two persons weighing circa 80 kg can increase this weight to 460 kg, i.e., by over 50%. The change in vehicle body mass $m_{s}$ also involves a proportional change in vehicle body moments of inertia $I_{sp}$ and $I_{sr}$. The experimental case mentioned above was not tested for the actual vehicle due to time limitations and limited use of the diagnostic station. However, due to the availability of the implemented vehicle simulator, it was possible to simulate this configuration. The absolute values and phase shift of the resultant transfer functions were compared with those related to the reference vehicle model and are presented in Figure 13 and Figure 14.

A comparison of the frequency characteristics of the absolute transfer functions indicates a significant and proportional decrease in amplitude for the vehicle model with passengers compared to the unloaded vehicle. This decrease is caused by the fact that similar forces generated by MR dampers move the increased vehicle body mass. Since both control-related signal paths simultaneously influence the response of the vehicle model, it is expected that the considered vertical acceleration of the middle vehicle body is decreased approximately two-fold. The increase in vehicle body mass also leads to a significant decrease in the first resonance frequency from 1.8 to 1.4 Hz for both the right and left vehicle parts.

The second group of figures presents a comparison of the phase-shift frequency characteristics. Here, it should be noted that the proposed identification method is dedicated to future applications in FxLMS for vibration control in the experimental vehicle, where the properly modelled control-related signal path has a critical influence on the convergence of the adaptive algorithm. According to [22], in order to maintain the convergence of the FxLMS under some classical assumptions, the phase error between the model of the control-related signal path and its actual dynamics should not differ by more than 90${}^{\circ }$ for any analysed frequency. It can be stated, based on the presented phase characteristics, that the requirement mentioned above is met in the case of a change in vehicle body mass. The greatest changes in phase shifts of the right and left suspension parts were equal to 63${}^{\circ }$ and 61${}^{\circ }$, respectively, for the loaded vehicle with a frequency equal to 1.2 Hz, which is close to the limit frequency of the analysis. Furthermore, the phase-shift change obtained for a frequency equal to 1.4 Hz, corresponding to the first resonance peak (loaded vehicle), is equal to 40${}^{\circ }$ for both parts of the vehicle. Thus, the control-related models obtained as a result of the presented identification procedure, which was conducted in the laboratory, can be applied to adaptive suspension control for a wide range of varying vehicle masses, especially during test drives.

## 5. Conclusions

The presented study reports a method for identifying control-related signal paths dedicated to an experimental off-road vehicle with MR dampers. The identified models describe the influence of forces generated by front suspension MR dampers on acceleration measured in the middle front part of the vehicle body. The identification goal discussed is not trivial since the MR damper, which is an example of a semi-active damper, cannot add vibration energy to the mechanical system but rather dissipates it. Thus, the main difficulty of this goal comes from the fact that the semi-active suspension needs to be simultaneously subjected to road-induced excitation and electric currents supplied to suspension MR dampers, while a response signal needs to be decomposed into road-related and control-related components.

During experiments, the front wheels of the all-terrain vehicle were subjected to a sinusoidal vibration excitation of a frequency equal to 12 Hz using a dedicated diagnostic station and specialised mechanical exciters. The harmonic type of road-related excitation allowed for its straightforward filtering from identification signals. Additionally, the front suspension MR dampers were controlled using a wide-band random signal with a 25 Hz bandwidth, different realisations, and several configurations, which differed in the average values and deviations of control currents. The measurement signals used for identification were taken from numerous sensors available in the vehicle, i.e., the MEMS accelerometer, suspension force and deflection sensors, and sensors of electric currents, which control the instantaneous damping parameters of MR dampers.

The final identification procedure needed to decompose the vehicle vibration response into components related to the forces generated by the right and left MR dampers, since the simultaneous control of both MR dampers was applied. The identification results evaluated in the frequency domain revealed several resonances of the vehicle response, e.g., related to the vehicle suspension and wheels, and their dependence on the selected configurations of control currents. Additionally, the parameters of the vehicle model with MR dampers and the diagnostic station were estimated based on the identification results. The analysis of simulation results of the implemented vehicle model carried out in the frequency domain showed the significant influence of the vehicle load on the absolute values of control-related signal paths and less influence on their phase shifts. The latter indicates that control-related models obtained as a result of the presented identification procedure, which was carried out in the laboratory, can be applied to wide range of varying vehicle masses, especially during test drives.

Adaptive vehicle suspensions are favoured for their ability to quickly adapt to varying road conditions and vehicle parameters. The identified control-related models of the semi-active suspension can be integrated into the adaptive suspension control algorithm in future research studies. The knowledge obtained from such models is crucial and required, inter alia, in order to maintain the convergence of the FxLMS, for which the phase error between the model and the actual dynamics should be kept within an acceptable range. Other future studies can focus on the extension of the presented vehicle model and the experimental analysis of the influence of different vehicle parameters on control-related signal paths.

## Figures and Tables

**Table 1:** Estimated parameters of the implemented Bouc–Wen model.

[$\gamma_{bw,0}$, $\gamma_{bw,1}$, $\gamma_{bw,2}$, $\gamma_{bw,3}$] = [12.490, 440.717, 1153.052, −564.019]
[$\lambda_{bw,0}$, $\lambda_{bw,1}$, $\lambda_{bw,2}$, $\lambda_{bw,3}$] = [10.898, −1.076, −4.102, 2.100]
[$c_{bw,0}$, $c_{bw,1}$, $c_{bw,2}$, $c_{bw,3}$] = [885.609, 130.047, 2009.233, −1342.062]
$n_{bw}$ = 2 $\epsilon_{bw}$ = 4.8

**Table 2:** Estimated parameters of the vehicle vibration model.

Mechanical vibration exciters			
$A_{rfb}$ = 0.002 m	$m_{efr}$ = 85 kg	$m_{efl}$ = 85 kg	
$k_{re}$ = 160,000 Nm${}^{-1}$	$c_{re}$ = 50 Nsm${}^{-1}$	$k_{ge}$ = 80,000 Nm${}^{-1}$	$c_{ge}$ = 50 Nsm${}^{-1}$
Vehicle wheels and tires			
$m_{ufr}$ = 10 kg	$m_{ufl}$ = 10 kg	$m_{urr}$ = 15 kg	$m_{url}$ = 15 kg
$k_{tfr}$ = 75,000 Nm${}^{-1}$	$k_{tfl}$ = 110,000 Nm${}^{-1}$	$k_{trr}$ = 70,000 Nm${}^{-1}$	$k_{trl}$ = 70,000 Nm${}^{-1}$
$c_{tfr}$ = 87 Nsm${}^{-1}$	$c_{tfl}$ = 126 Nsm${}^{-1}$	$c_{trr}$ = 102 Nsm${}^{-1}$	$c_{trl}$ = 102 Nsm${}^{-1}$
Vehicle body and suspension damping			
$m_{s}$ = 300 kg	$I_{sp}$ = 75 kgm${}^{2}$	$I_{sr}$ = 33 kgm${}^{2}$	
$l_{f}$ = 0.543 m	$l_{r}$ = 0.607 m	*w* = 0.135 m	
$c_{usfr}$ = 300 Nsm${}^{-1}$	$c_{usfl}$ = 300 Nsm${}^{-1}$	$c_{usrr}$ = 300 Nsm${}^{-1}$	$c_{usrl}$ = 300 Nsm${}^{-1}$
Shock absorbers and suspension design			
$k_{safr}$ = 22,208 Nm${}^{-1}$	$k_{safl}$ = 22,208 Nm${}^{-1}$	$k_{sarr}$ = 34,857 Nm${}^{-1}$	$k_{sarl}$ = 34,857 Nm${}^{-1}$
$g_{z_{saf}}$ = 0.515	$g_{z_{sar}}$ = 0.428	$g_{F_{saf}}$ = 0.549	$g_{F_{sar}}$ = 0.501
$\alpha_{saf}$ = 67${}^{\circ }$		$\alpha_{sar}$ = 60${}^{\circ }$	
Harmonic oscillator included in vehicle model response ${\hat{a}}_{sf}$			
$\omega_{\mathrm{res}}$ = 2π×5.5 Hz	$\zeta_{\mathrm{res}}$ = 0.04	$g_{\mathrm{res}}$ = 8.42 × 10${}^{-4}$ kg${}^{-1}$	

## Data Availability

The data presented in this study are available on request from the corresponding author.
